# Hepatocellular carcinoma arising from adenoma with ARID1A mutation in an adolescent patient with ATM germline mutation

**DOI:** 10.1093/jscr/rjaf835

**Published:** 2025-10-26

**Authors:** Elizabeth Drda, Samira Ali, Haind Fadel, Belinda Sun, Walaa Elfar

**Affiliations:** Department of Pediatrics, Banner - University Medical Center Tucson, Pediatrics, 1501 N Campbell Ave, Suite 3335, Tucson, AZ 85724-5573, United States; Department of Pediatric, Division of Gastroenterology, Banner - University Medical Center Tucson, Pediatrics, 1501 N Campbell Ave, Suite 3335, Tucson, AZ 85724-5573, United States; Department of Pathology, Banner - University Medical Center Tucson, Pediatrics, 1501 N Campbell Ave, Suite 3335, Tucson, AZ 85724-5573, United States; Department of Pathology, Banner - University Medical Center Tucson, Pediatrics, 1501 N Campbell Ave, Suite 3335, Tucson, AZ 85724-5573, United States; Department of Pediatric, Division of Gastroenterology, Banner - University Medical Center Tucson, Pediatrics, 1501 N Campbell Ave, Suite 3335, Tucson, AZ 85724-5573, United States

**Keywords:** pediatric hepatocellular carcinoma, hepatic adenoma, ATM mutation, ARID1A mutation

## Abstract

While hepatocellular carcinoma (HCC) is increasingly common in adults, it remains rare in children. Unlike adult HCC, which typically arises from cirrhosis, pediatric cases often occur without underlying liver disease. We report a 14-year-old male with a large hepatic adenoma containing multiple foci of HCC. Genetic testing revealed a germline ATM mutation and a somatic ARID1A mutation. We hypothesize that impaired DNA repair from the ATM mutation facilitated the ARID1A mutation, driving malignant transformation. This case underscores the importance of recognizing malignant potential in hepatic adenomas, utilizing appropriate imaging and pathology, and monitoring for associated cancers in genetically predisposed patients.

## Introduction

Hepatocellular carcinoma (HCC) is a rare pediatric malignancy with poor overall survival and a male predominance. Together, HCC and hepatoblastoma represent only 0.5%–1.5% of childhood cancers, with ~4% occurring in pediatric liver transplant recipients [1, 2] Hepatoblastoma accounts for ~80% of pediatric liver cancers; HCC comprises the remaining 20% [1, 2].

Unlike adults, pediatric HCC often arises without cirrhosis, linked to metabolic, infectious, vascular, or idiopathic causes [3–5] Malignant transformation of hepatocellular adenomas (HCAs) is rare in children, with a meta-analysis showing transformation in 4.2% and focal malignancy in 4.5% of resected HCAs [6].

We present a rare case of HCC arising from a large hepatic adenoma in a child, associated with an ARID1A mutation—a tumour suppressor gene implicated in several cancers, including clear cell ovarian carcinoma, gastric adenocarcinoma, and cholangiocarcinoma [7–9]. This report highlights the clinical presentation, diagnostic workup, and the value of a multidisciplinary approach in pediatric HCC.

## Case report

A healthy 14-year-old male presented with acute right upper quadrant abdominal pain, moderate distension, nausea, and bloating. He had no prior abdominal symptoms, systemic complaints, or relevant medical/family history, including liver disease or viral hepatitis.

**Figure 1:**
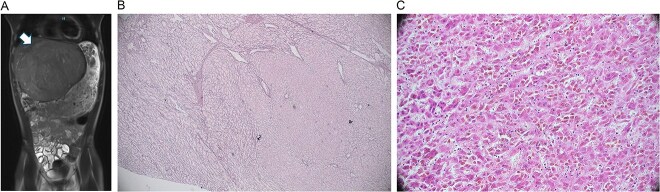
Radiology and pathology findings from a 14-year-old male with hepatocellular carcinoma (HCC). (A) MRI abdomen/pelvis with large, encapsulated liver mass (arrow) and associated mass effect. (B) Reticulin stain revealed the adenoma component with an intact staining pattern at the periphery and the HCC component with loss of staining at the center. (C) High power haematoxylin and eosin stain of hepatocellular carcinoma.

On exam, his abdomen was soft but distended, with RUQ tenderness. The liver edge was palpable 4 cm below the ribs; the spleen tip was also palpable. Computed tomography (CT) abdomen/pelvis revealed an 18 × 15 × 14 cm left hepatic mass compressing adjacent structures. Labs showed mild microcytic anemia, thrombocytosis, elevated PT/INR, and undetectable alpha-fetoprotein. Magnetic resonance imaging (MRI) showed a large, well-encapsulated, T2 hyperintense, T1 hypointense mass with a central scar, suggestive of focal nodular hyperplasia (Fig. 1A). Biopsy indicated a mutated adenoma with high malignant potential.

Arterial embolization and partial hepatectomy were performed. Pathology revealed a 19 cm mass with multiple nodules and a central scar. Microscopy showed multiple foci of moderately differentiated HCC within the adenoma (Fig. 1B and C), with loss of reticulin, CD34 vascular staining, and glypican 3 positivity. The tumour was staged as pT1b, with negative margins and no metastasis on chest CT.

Genetic testing identified ARID1A and ATM mutations, with ATM also present in the germline. Additional variants of unknown significance included BRCA1, GNAS, KDM2B, MSH6, and HSP90AA1.

Follow-up imaging at 6- and 12-weeks post-op showed no recurrence. Surveillance MRI/CT every 6 months and labs every 3 months have remained stable and disease-free.

## Discussion

Pediatric HCC is rare, with an incidence of 0.4 per million in children under 14 and 1.4 per million in adolescents [10]. It accounts for just 0.4% of all HCC cases [11]. Unlike adult HCC, which typically arises from cirrhosis, ~70% of pediatric cases occur without underlying liver disease [10]. Hepatic adenomas are also rare in children (<5% of hepatic tumours), often linked to hormonal imbalances or metabolic disorders [12]. Malignant transformation into HCC occurs in ~4.2% of adenomas, especially in males and tumours >5 cm [13].

In this case, we propose that the patient’s germline ATM mutation and tumour-specific ARID1A mutation contributed to adenoma formation and malignant transformation. ATM, a DNA repair gene, is associated with various cancers but not previously linked to HCC [14]. ARID1A, a chromatin remodeling gene, is implicated in multiple malignancies including HCC and cholangiocarcinoma [15]. We hypothesize that ATM-related DNA repair deficiency enabled the ARID1A mutation, driving tumourigenesis.

ARID1A loss disrupts chromatin architecture and gene regulation, promoting metastasis [16]. ATM deficiency impairs DNA repair, increasing genomic instability. Together, these mutations may synergistically enhance oncogenic potential. This dual profile may also inform treatment, as ARID1A-deficient tumours respond to DNA-damaging agents and immune checkpoint inhibitors, while ATM loss may sensitize tumours to PARP or ATR inhibitors.

This case underscores the importance of timely diagnosis and recognizing malignant potential in large hepatic adenomas, especially with underlying genetic mutations. Initial imaging suggested focal nodular hyperplasia, but pathology revealed HCC within an adenoma, highlighting the need for multidisciplinary evaluation.

## Conclusion

This rare pediatric case of HCC arising from hepatic adenoma with ATM and ARID1A mutations underscores the importance of genetic evaluation, multidisciplinary diagnosis, and long-term monitoring for associated malignancies.
